# Futureproofing triathlon: expert suggestions to improve health and performance in triathletes

**DOI:** 10.1186/s13102-019-0153-5

**Published:** 2020-01-10

**Authors:** Michael D. Kennedy, Camilla J. Knight, Joao Henrique Falk Neto, Katie S. Uzzell, Sara W. Szabo

**Affiliations:** 1 Athlete Health Lab, Faculty of Kinesiology, Sport, & Recreation, University of Alberta, 4-230 Van Vliet Complex, Edmonton, AB Canada; 2 School of Sport and Exercise Sciences, Swansea University, Swansea, UK

**Keywords:** Knowledge translation, Sport science, Coach education, Sport performance, Athlete health

## Abstract

**Background:**

Given the multi-modal nature of triathlon (swimming, cycling, running), training for a triathlon event has numerous potential health benefits including physical fitness. However, triathletes also have a high prevalence of health issues including overuse injury, illness, fatigue, and burnout. To address the ongoing prevalence of health issues, roundtable discussions were organized at the International Triathlon Union Science of Triathlon 2017 conference to develop strategic objectives deemed necessary to “futureproof triathlon”. Futureproofing as a concept serves to design new approaches and ways of thinking to reduce consequences in the future. In this case, the futureproof process aimed to develop key recommendations for triathlon.

**Methods:**

This qualitative study had 22 participants including athletes, coaches, practitioners, academics, and policy makers who participated in roundtable discussions at the Science of Triathlon conference. Seven of these participants completed follow-up semi-structured interviews on the same topics. The data collected from the roundtable discussions and the semi-structured interviews was analyzed using thematic analysis.

**Results:**

Five main themes were produced: “Critical appraisal and application of knowledge”; “Integrated approaches to developing, disseminating, and using research and expertise”; “Appropriate development and use of measures for monitoring training and recovery”; “Knowing your athletes and adopting holistic approaches to athlete/person-development”, and; “Challenging accepted cultural and sporting norms”. Participants indicated the need to reduce the knowledge gap between research and practice as well as a more collaborative approach to triathlon research development amongst coaches/practitioners and academics. It was stated that current monitoring tools require more research to determine which are most useful to informed decision making for coaches/practitioners. It was cautioned that data driven assessments should be used judiciously and be athlete centered. Triathlon as a sport should also have a greater focus on healthy participation and development of youth athletes.

**Conclusions:**

A series of applied implications were developed based on these five themes as guiding principles for how to futureproof triathlon. Additionally, roundtable and interview participants who held varying positions and opinions within the sport of triathlon agreed that the unique challenge of training for and competing in a triathlon should not be forgotten in the futureproofing of the sport.

## Background

The “triathlon”, a sport that combines swimming, cycling, and running disciplines into one event, has always been viewed as challenging to the human body and spirit [1]. The unique physical challenge of three distinct modes of exercise packaged into one event is key to its past, current, and future popularity. In fact, since its inception in the early 1970’s the sport of triathlon has flourished to over 4 million competitors annually in North America alone. However, the commitment to training for swimming, cycling, and running concurrently means triathletes may incur more hours of sport-specific training than a single mode athlete [2–4]. In fact, the amount of training some triathletes accumulate is substantial, with research showing that a world-class female performer completed 796 sessions in the 50 weeks (approximately 16 training sessions per week) leading up to the 2012 London Olympics [3]. This frequency allows the necessary training within each mode to produce a training effect but is greater than double mode athletes such as Nordic skiers (11 sessions per week) [5] or single mode athletes such as runners (12 female or 13 male sessions per week in world class marathoners) [6].

Given such training loads it not surprising that triathletes incur significantly more overuse injuries [7, 8], severe over-reaching [2], overtraining [9], and illness [10] compared to other endurance sports. This has led some authors to conclude that triathlon training leads to negative health outcomes [10] or unhealthy behaviors while pursuing triathlon training goals [1]. Furthermore, there is a lack of evidence indicating that triathletes commit time to training other aspects of fitness, particularly muscular strength, which has long been known to improve endurance performance [11] and reduce risk of overuse injury in athletes [12]. Thus, available research indicates that: a) triathletes acquire significant total training stress due to the concurrent training of the disciplines; b) increased total training stress affects triathletes’ health, well-being, and incidence of overuse injury, and; c) extensive sport-specific training may lead to cumulative stress and increased injury risk [9], thus triathletes may benefit from improved health and fewer overuse injuries with a greater focus to training other components of fitness such as muscular strength.

Given these facts, the International Triathlon Union (ITU) nominated Edmonton as the site to host the 2017 Science of Triathlon Conference with a special focus on the health and well-being of triathletes. The conference included a select group of internationally acclaimed researchers whom have studied the psychology, physiology, medical issues, developmental issues, and environmental issues associated with triathlon, endurance, and elite sport. As part of the conference, the organizers, in concert with the ITU, held scientific roundtables that brought together the scientific presenters with high-level coaches, athletes, and policy makers with the focus, “what are the current issues around triathlete health and wellbeing and how do we plan for improved health and wellbeing?”. These sessions were collectively called “futureproofing triathlon” and were recorded to produce a digital archive for conference attendees and triathletes worldwide. The term futureproofing has been defined as “the process of anticipating the future and developing methods of minimizing the effects of shocks and stresses of future events” [13]. Given this definition, we felt that the concept of futureproofing captured both the lack of scientific evidence regarding strategies to improved health and performance as well as the spirit of the conference objectives. In the context of this futureproof definition this paper aims to provide recommendations that serve to a) improve current challenges in triathlon health and performance and b) provide a framework to address future stresses and challenges in the development of triathlon as a sport. Specifically, these recommendations serve as a key step towards a new vision of health, well-being, and improved performance in the sport of triathlon as well as contributing to the body of triathlon related research.

## Methods

### Methodology

There were two stages to our methodological approach, both of which were underpinned by a qualitative descriptive approach [14]. Firstly, a qualitative approach was deemed most appropriate because it ensured that we could obtain a range of individual’s insights and perceptions. It ensured we could draw on a broad range of experiences and limit the imposition of any pre-existing categories or ideas on the participants. Secondly, the reason for combining round table discussion with interviews was to maximize the collection of naturalistic data from a range of experts through the panel discussions and then provide an opportunity to gain further insights and reflections in the follow up interviews. In the first stage, four “futureproofing” roundtables were conducted with 22 individuals, including researchers, coaches, athletes, academics, and policy makers from Canada, England, France, Japan, Norway, Scotland, South Africa, Spain, United States of America and Wales. These futureproofing sessions provided an initial opportunity for individuals to share their unique perceptions with their intrinsic knowledge regarding the future of triathlon and the necessary focus to enhance the health, well-being, and performance of athletes, while also seeking consensus across the panel. The combination of panelist’s ensured a broad spectrum of topics was considered and discussed. The second stage of the study occurred through individual semi-structured interviews with seven individuals who participated in the initial futureproofing sessions (nations represented were: 3 Canada, 1 France, 3 United Kingdom). The purpose of these interviews was two-fold: to provide an opportunity to gain feedback on the themes that had been identified from the roundtable discussions and to seek additional insights into areas deemed necessary for futureproofing triathlon. Through these individual interviews, explicit suggestions to address potential challenges associated with the future of triathlon were also sought. Thus, this two stage approach was utilized to provide an opportunity for panelist’s to provide further reflections on the data from the panel discussions and seek clarity around ideas and interpretations that were being developed through the initial phase of data analysis.

### Participants and procedure

Keynote speakers, high-performance coach attendees, elite athletes, and triathlon policy makers were contacted prior to the 2017 ITU Science of Triathlon conference to ask if they were willing to participate in futureproofing expert panel discussions. If they agreed, participants were asked if they would consent with the information shared in these sessions being used as data to underpin the development of a paper on futureproofing triathlon. Following agreement, the panels were selected, and the sessions were scheduled throughout the conference.

Each panel comprised between five to seven individuals, including a chair that was responsible for asking questions and facilitating discussions. In total, 22 individuals took part in the panel discussions. This included ten academics (two academics participated in two panels) whose research areas included altitude/hypoxic endurance training; training stress; endurance athlete health; motivation and determinants of psychological success in endurance performance, positive youth development in sport, talent development in sport, health risks of endurance training and competition; environmental considerations in training and competition of endurance sport; and government sport policies. Four coaches who worked with Olympic, elite, and age-group triathletes, one athlete, and six policy makers including the members of the ITU board, the ITU Medical Committee and one policy maker who was the head of one of the largest national federations in the ITU. There was also one industry representative from the pre-eminent online digital training log software company. This individual contributed insight on the monitoring of training and athlete status and how industry and triathlon can collaboratively work together.

The questions were pre-determined before each panel based on the proposed topic for that panel. Topics included understanding training and competition stress in triathletes, medical consequences of training and competition in triathletes, improving talent development decision-making and retention of youth triathletes in the sport, and how federations can improve and support change in the futureproofing of triathlon. During the actual panels, there was flexibility within the discussions to enable participants to lead discussions based on previous comments and also to allow for audience questions to be addressed. Each panel discussion lasted approximately 45 min and was video recorded for future circulation and analysis.

Following completion of the first stage of the study, all individuals who participated in the futureproofing sessions were contacted via e-mail and invited to participate in follow-up semi-structured interviews. Along with the invitation email, all individuals were also sent the initial themes based on the first stage of the study. A secondary reminder was sent to all panelists if we did not hear from them after the initial e-mail. Seven participants from the futureproof sessions agreed to participate in a follow-up interview. Individuals who were not available for interview were asked to provide comment on the initial findings via email, however no feedback was provided. The semi-structured interviews were scheduled at a time convenient to the participant and took place either via skype or in person (depending upon the geographical location of the participants).

The interview guide was developed based on the themes that were derived from the stage one panel discussions. Thus, the interview guide included five main questions corresponding with the five main themes discussed during the futureproofing panel discussions: appraisal and application of knowledge, use of available research expertise, monitoring training and recovery, adopting holistic approaches to athlete/personal development, and challenging accepted dogma and sport culture norms. The goal of the questions was to allow each participant the opportunity to elaborate on the theme and provide strategies for implementation within triathlon. Thus, each theme was briefly described before participants were asked to share their views on the theme. Follow-up probes were used as necessary to obtain further insights. Interviews ranged in length from 51 min to 98 min (mean = 76 ± 17 min).

### Data analysis

Both the panel discussions and the semi-structured interviews were analyzed through a process of thematic analysis [15, 16]. Analysis of the futureproofing discussions occurred first, followed by the collection of the semi-structured interview data and subsequent analysis. Thus, it should be noted that while the analysis of the futureproofing discussions was generally approached inductively, the analysis of the interviews was initially deductive (i.e., the transcripts were reviewed based on the themes from stage one) followed by a secondary inductive analysis (i.e., seeking to identify new themes and ideas).

The audio files of the roundtables were transcribed verbatim before being read repeatedly to search for meanings and patterns. Once familiar with the data the co-investigator then moved onto the production of initial codes. Having produced the initial codes, the co-investigator then moved onto systematically examining the collated data and filtering the codes in order to establish relationships between codes to develop potential themes. The process involved organizing relevant coded extracts within the main themes to form a set of candidate themes. These codes were then rearranged into main themes and subthemes dependent on their relationship. Next, the candidate themes were closely scrutinized to ensure they accurately represented the coded extracts. Any coded extracts that were not deemed coherent were either split, combined, or discarded. Further refinements of the specifics within the candidate themes and definitive and informative names were then developed for each theme and sub themes were also created. Following the creation of these final themes from the roundtable data, the semi-structured interviews were conducted and then analyzed. Data that fitted with pre-existing codes and themes were identified before “new” data codes were developed.

## Results

Through the process of data analysis, five themes pertaining to futureproofing triathlon were identified. Each theme, with accompanying sub themes is described below.

### Critical appraisal and application of knowledge

The first theme pertains to the importance of practitioners, coaches, and athletes ensuring they engage in critical appraisal and application of knowledge within triathlon. That is, it was perceived that for triathlon to develop it was necessary to ensure that practice was founded upon appropriately applied, critically appraised knowledge rather than only experience, conventional wisdom, or anecdotal data. For instance, an academic shared during a panel discussion:

This phrase around survivorship bias is how much of our current evidence is based on those athletes that have been successful. And how much do we really know about the train wrecks, the people where the wheels have fallen off, around the injuries that have contributed toward those watershed adolescent years where we lose a lot of our athletes between the ages of 12 to 16.

*Critically consuming literature from within and beyond triathlon* was seen to be particularly important if one was to start to apply knowledge in practice. When considering a critical approach to consuming knowledge, an awareness of methodological and contextual considerations (i.e., whether the papers were methodologically sound) was seen as particularly important. For instance, interview participant 3 shared:

It will never be as simple as looking at a paper in a journal, and say ‘okay I have to do one minute work interval is better than 20 minutes’, because you have to look at it on the context, you have to take into account what the next competition, the prioritization..

Similarly, the extent to which the studies have been conducted in a similar population which would influence their transferability into triathlon was deemed important. As interview participant 7 shared:

And so, going back to the origins of the question, scientists tend to deal with groups inevitably, for statistical reliability and validity. And it’s the differences between the athletes, as much as the similarities between the athletes, that will determine whether or not the coach is successful and whether the athlete fulfils their potential, or not.

Fundamentally, it was widely accepted that for triathlon to flourish and particularly for athletes to improve their performance as well as their health and well-being it was deemed important that practitioners and coaches did not just “blindly” accept findings.

Building upon this, it was also suggested that practitioners and coaches needed to *engage in continual informed debate regarding conventional wisdom.* That is, rather than just doing things because others did them or it had always been done in a certain way, it was perceived that reflection on individual practice was particularly important. Interview participant 6 suggested:

So I think when you’re looking at – for me I’m looking at swim, bike, run. Okay is there better ways to teach swimming. Bike, what are – we use power meters, is that a good thing? And then the running, running has such a history behind it where you have so much anecdotal … Knowledge vs. okay, this put them in laboratory and give them a Maximal Oxygen Consumption (VO2max), that kind of thing.

To facilitate such reflection, it was suggested that practitioners, coaches, and athletes would benefit from monitoring athletes’ engagement and track changes in their health, well-being or performance to enable identification of what might work for a particular athlete. As interview participant 1 suggested:

One of the key things for practitioners and for coaches is making sure when they are attempting to apply these, you know changes or things from research, it’s not instantly throwing out what you’re already doing. But it’s considering how this may work within your setting, what individual adaptations you might need to make; and then implementing it, trying it, seeing if it works for you.

### Integrated approaches to developing, disseminating, and using research and expertise

Recognizing the importance of (critically) utilizing research that pertains to triathlon, it was also identified that for triathlon to succeed in the future, it was important that an integrated approach to developing and disseminating research be developed. That is, the individuals highlighted the need for greater interaction between researchers, practitioners, coaches, and athletes to ensure that research being conducted addressed the “real world” problems being faced within the sport.

To enable such an integrated approach, it was suggested that *organizations working together* within and beyond triathlon was needed to share good practice and knowledge. As interview participant 2 stated:

I think certainly you can have people working together, but you have to have researchers that are open to see something else than what they study. So it comes down to the body of research and how good the researchers are, but it also comes down to the personality of the researcher and are they able to understand where other people are coming from and what perspective they’re bringing to the table?

By researchers and others working together it was perceived there would be greater buy-in from coaches, athletes, and practitioners to the research that is being conducted.

It was also thought that such buy-in would occur if the skills of all (i.e., the coaches’ various experiences, the athletes’ individual experiences, the researchers’ applied and research knowledge) were acknowledge and utilized. That is, rather than assuming researchers only know research and coaches only know practice, recognizing the contribution of each member is key. As highlighted by a practitioner-academic in a panel discussion:

… The importance of being open to what different people with different backgrounds can bring to the table. And then there is a need for scientists to appreciate what practitioners do and not see it as a black and white situation where we have these skills and they don’t have these skills, so that they don’t understand what we’re doing and why we’re doing what we’re doing. And then the practitioners have to also be open that there are a lot of scientists that are athletes, or past athletes, are coaches in their free time, they’re really passionate about sports, and it’s not ‘cause they work as a scientist during the day, that they are at a distance from the reality of sports.

Particularly, in seeking to recognize the contribution of all and produce a unified approach to research and practice, it was suggested that an *integrated approach to developing research questions and producing knowledge was needed*. It was thought that practitioners and researchers working together from the outset of a project would enable individuals to overcome the science-practice gap. As a practitioner suggested in the roundtable, “I think in an ideal world what we really want is we want coaches, practitioners, academics working together right from the start rather than us having to disseminate information, or you guys [coaches] having to bring information to life for yourselves.”

If such an approach was not possible, seeking *improved accessibility of research in practice* was also seen as valuable. As interview participant 4 said:

I guess knowledge translation would be a big, big piece of that, for sure, and you see a lot of it in terms of social media and stuff. I think of, there’s these YouTube channels the Global Triathlon Network and the Global Cycling Network.

By drawing on more creative and different ways to disseminate research findings, it was anticipated that more coaches/practitioners would be able to access and use research. Further, the simplification of language to provide clearer guidelines for practitioners and coaches were deemed important. One academic summarized in a roundtable, “I think to me probably the most important thing it’s simplifying the language and making things accessible as much as possible.”

Finally, participants suggested that *seeking and accessing opportunities to learn from and develop alongside others* was important to enable greater understanding and application of research and expertise in practice. Specifically, greater organizational support for coach development courses as well as enhanced facilitation of knowledge sharing opportunities were thought to be particularly needed and beneficial. Interview participant 5 shared:

… wouldn’t it be nice if the race organizers of triathlons would have a mind towards facilitating and inviting research opportunities. And some of it may or may not be compatible with the population or the race itself, but it would be nice if all of the dozens and dozens, if not hundreds of triathlon events had a research mandate and they could perhaps generate a call for research proposals that would populate their races, they could easily recruit scientists and integrate them in a more fluid way to existing events.

### Appropriate development and use of measures for monitoring training and recovery

Moving away from research application, the next theme focused upon the increasing trend towards athlete monitoring within triathlon. It was suggested that to futureproof triathlon, the development of more appropriate measures, as well as more appropriate use of measures to facilitate monitoring of training and recovery, were needed.

Firstly, participants raised questions pertaining to the *appropriateness of the data* that is currently obtained. Specifically, participants suggested that for data to be useful moving forwards, coaches and practitioners must be seeking to integrate the person within the data (i.e., recognize the range of challenges and demands beyond the data that may be impacting on an individual). As interview participant 2 said:

There should be room for some measuring of more of the emotional cognitive states of the athletes and how they feel about their training. So it’s not as objective, like there’s no hard data that you can kind of measure it directly. But in using those monitoring systems, we should try to leave some room so that the athletes are able to record … , the quality of their relationship with their coach or their team mates, and maybe even track their – their mood over a training or a season.

In fact, it was suggested that although the current trend is moving towards a reliance upon “objective” data measures, the future of triathlon and particularly the health and well-being of athletes would be enhanced if this was reduced and instead coaches/practitioners focused more upon communicating with athletes and learning how they are feeling from them. As one coach shared in a roundtable, “We all need to remember that they [monitoring systems] can distance the coach from the athlete and they should never replace very close regular communication.”

Participants also questioned the *necessity of the data* that is currently collected, suggesting that data is collected without a clear reason. Interview participant 1 suggested:

And I think it’s really great to monitor, but I think there has to be a purpose to it, because otherwise what’s the point in doing it? I think people get really caught up in the process, so I agree I think monitoring different things can be really helpful, but I think then it has to be individualized to allow you to see what’s going on for that athlete.

Participants suggested that there is an increasing push towards practitioners and coaches collecting data just to “have it.” However, they suggested that unless data were being collected to inform coaching practice of individual athletes (which was time consuming and challenging at times), there was limited reason to collect it. Interview participant 5 explained:

My cautionary note to athletes would be, use the stuff [monitoring data] very judiciously and then it should ***help*** – and I would put that in italics and underline and boldface, ***it should help*** inform how you feel, and it helps you towards a healthier more productive training experience.

Rather, participants suggested that, to futureproof triathlon, it was necessary that coaches and practitioners *individualized their application and use of data*. That is, ensuring that they took care to fully understand each of their individual athletes and know what any data collected means in relation to them. As one coach shared, “The three of us need to be a team to come up with a plan. Don’t be afraid to communicate with your athlete, but also any kind of other support staff around the athlete, cause the communication and individualization is key.” Further, it was suggested that coaches and practitioners should attend to the specific needs of their athletes when seeking to collect data (i.e., choosing what data to collect, how much to collect), as interview participant 6 stated, “Yeah for sure, because the coach can check how the understanding of okay what’s the understanding of the athlete. Or physiology, biomechanics, psychology, race feedback.”

In individualizing and applying such data, it was, however, recognized that some consideration of the *accuracy of data collected* was needed. Particularly, it was deemed important that coaches, practitioners, and athletes themselves understood the limits of different technological measures and the errors associated with them. For instance, one academic shared, “They’ve been using heart rate viability and with quite a good success I would say … but I think the interpretation, the analysis, is a bit more tricky … a lot of methodological issues to be sorted out.” Particularly, it was highlighted that the sensitivity of measures needs to be recognized and steps taken to ensure that measures are more sensitive, valid, reliable, and most importantly simple and quick. As a coach shared on one roundtable:

So in a sense to me a thorough measure of rest and recovery is probably the most appropriate metric if you like, rather than a concern about the training stress scores on any of those individual disciplines that you mentioned earlier. So my question would be really around what can we do to accurately, reliably, validly measure recovery.

In addition to an awareness of, and need to overcome, technological limitations of monitoring equipment and data, some *ethical and personal considerations* were also identified. Particularly it was suggested that greater consideration pertaining to who owns athlete data when it is collected is considered, as an academic shared in a roundtable:

But then we run into some challenges that are equally important with that technology. Ethics, for example, who owns the data? where does it go? what do you do with it? who do we ask permission to use this data and make all these analyses? We can actually go quite far in predicting behavior with this type of data, have we actually asked for permission to do so?

### Knowing your athletes and adopting holistic approaches to athlete/person-development

Linked to the aforementioned coach-athlete considerations and the need to individualize data to each athlete, participants highlighted that the future of triathlon both with regards to athlete health and performance, requires coaches and practitioners to commit time to knowing their athlete as an individual and as a person. As one coach simply stated in the panel discussion, “It has to be athlete-centered, or I heard earlier person-centered, which even feels better that our main people are at the center of our work, and for all of us that’s, of course, the athletes.” To do this, it was suggested that coaches *commit time to listening to and learning from their athletes* allowing them to individualize their work, as an academic shared on the panel, “So I think coaches, if you’re listening that is helpful, and the comment about the 3-way conversation between the coach, the athlete, and the medical supports staff, and it should be a 3-way conversation.”

Particularly important was that coaches take time to *understand their athletes’ motives* and account for the changes in this across the lifespan. Thus, adopting a developmentally-appropriate approach to different athletes to facilitate sporting development and performance was key. As interview participant 3 shared, “So there’s, there’s a definite progression from okay you’re a 12 year old and coach led. Okay. All the way up to, and it’s – it’s not going to be coach led program all the way.”

Alongside this, it was suggested that coaches commit *to understanding athletes’ broader lives* and recognize how this may have an impact on training/competition. A coach shared:

I come from the age-group athlete world and so with that the life stress is also a huge component to that. So to be able to take that into account where we’re not measuring it through a device but it’s still having an impact on not only their training and their ability to train but how they’re responding to it.

A key consideration within this was to ensure that coaches integrated family and broader support network within their work, to *develop a team approach to support*. As participant 7 suggested:

So it’s almost important that there’s a third party involved, who helps guide the decision making, who isn’t the coach, and who isn’t the athlete, who can say well if you got to A, they offer this, but they don’t offer that.

### Challenging accepted cultural and sporting norms

Reflecting on the landscape of triathlon, participants reflected on the current system and highlighted that there are some issues within the system. Notably, participants indicated that some of the current practices within triathlon are simply accepted norms, which go unquestioned. For instance, interview participant 1 said:

So often these things develop because at the time they made sense, but then you have to say “well does it still make sense now?” So you know when there’s arguments made about you know athletes being certain weights, and then you look at athletes and they’re all sorts of shapes and sizes. Okay well now does that norm still hold true?

To overcome limitations or perceived failures within the current system, it was suggested that coaches and practitioners should focus on the creation of a safe, developmentally appropriate environment for long-term engagement and success. As interview participant 2 shared:

Sure it takes a long time to become an elite level athlete, but why do we have to design sporting systems to develop elite level athletes only? If we have young kids involved in sport, we should think about short term athlete development. And we should think about, you know, as an adult it’s nice to think long-term, sure it takes 15-years to become an elite level athlete, but the 12-year-old that’s in your practice today, doesn’t think about 15 years from now. She thinks about tomorrow, and she thinks about after practice today. So, so the short-term experiences that athletes get out of sport, accumulate over time and create this environment.”

Particularly, it was argued that the greatest progress would occur if coaches *resist the monetarization of youth sport*. Further, it was suggested that *minimizing outcome/performance focus* and instead *emphasizing fun* was important, as a coach shared on a panel:

So there’s a fair number and all I’m gonna say is keep it fun. It’s really simple. There’s no measurement for it, unless you ask how much fun are you having guys? And that again is just for the longevity as well in keeping kids healthy, keeping them active, and keeping them in sport. If they’re enjoying what they’re doing, there’s gonna be a longevity in the life of their sport.

To do this, encouraging a reduction in early specialization and a focus on multi-sports was purported as important to reduce the focus on performance and outcomes. As one academic shared in a roundtable, “We are too often treating them [children] like mini adults and we are suffering perilously because we aren’t designing programs and offering experiences that are developmentally appropriate.” An athlete also shared, “And not just in sport, learning teamwork and how? it branches outside of sports as well, just being good people, how to interact socially, and all that kinda thing. And then also me coming through into triathlon.”

## Discussion

### Overview

The purpose of this paper was to provide recommendations that serve to first and foremost improve current challenges in triathlon health and performance. Second, we took a futureproof approach as a means to accommodate future stresses and challenges in triathlon. Specifically, it is hoped that these recommendations are a key step towards a new vision of health, well-being, and improved performance in the sport of triathlon. Drawing from expert roundtable discussions and semi-structured interviews, five themes were developed which are discussed in relation to existing literature below along with specific recommendations for coaches, practitioners, and academics.

The systematic manner in which we gathered and analyzed the information via roundtables and interviews hopefully provides sound “evidence” to promote change in triathlon. It has been previously stated that “using evidence” is an important aspect of improved sport outcomes and coaching effectiveness [17], where the use of evidence leads to “correct decision” making in coaching [18]. Thus, the findings from this study reinforce that expert coaches and practitioners working with triathletes understand that the use of evidence-based information can improve coaching effectiveness and are aware of its importance to improve decision making. This is a positive step in futureproofing the sport of triathlon as it has been demonstrated that coaches’ motivation to find new ideas is key in enhancing their practice [19].

### Application of findings

In reference to theme 1 it was clear that concerns still exist in how coaches and practitioners access relevant evidence and how that evidence is critically appraised. Previous research on coaching has demonstrated that coaches often seek knowledge from formal (coach education opportunities) and informal sources (interactions with other coaches, own experiences and reflections, experience with athletes) with informal evidence gathering being more common [19, 20]. In general, informal evidence is defined as “unguided or incidental” [21] and viewed as efficient knowledge acquisition and effective at informing decision making compared to formal education [22]. Although informal evidence gathering is preferred, similar to the suggestion from the roundtables/interviews, coaches value evidence-based information but see finding this information as a major barrier to accessing it [20]. In this context, improved access to quality evidence relevant to triathlon is a pertinent futureproof objective.

To borrow from research that looked at how to improve concussion education delivery for sport coaches and parents, the authors of that project suggested the following regarding evidence gathering: “make materials user-friendly, interactive, and utilize more than one modality to present information” [23]. Similarly, it has been suggested that the development and presentation of evidence in a manner that is user-friendly and accessible in different modes (print, digital, podcasts) is key to communication of such information to coaches [20, 24]. In addition, a further necessity is the integration of sport science within organizations [24, 25]. Indeed, lack of a direct access to a sport scientist has also been listed as one of the key barriers for coaches to access sport science knowledge [20, 26]. In fact, improved access via the aforementioned points would likely lead to improved “evidence-based practice” for triathlon coaches considering that “best relevant research” has been identified as one key aspect of current thinking on how to best approach evidence-based decision making [17].

Theme 2 builds on theme 1 where panelists also acknowledged in theme 2 that improved interaction between coaches, researchers, and sport scientists would improve the apparent “knowledge – practice gap” [20] that currently exists in triathlon. Martindale [25] investigated reasons why published research is not consistently being used to improve practice in the sport, finding that many coaches perceive that research is not relevant to them. One suggestion that would address both the Martindale [25] findings and the panelists’ expert opinions is through an improved process of collaboration between researchers/academics and coaches/practitioners. Specifically, involving both researchers/academics and coaches/practitioners in the development of research from the outset would improve the relevancy of the research to triathlon. This has been previously advocated as an effective strategy in developing new research/evidence that could be more useful to coaches and practitioners in informing practice [17, 24, 27] and is being effectively utilized in other organizations (e.g., The Welsh Institute of Performance Science, Wales, United Kingdom). In fact, sport science research, including that in the sport of triathlon, has been questioned as not being real-world because too much of the research occurs in a controlled lab over a short duration [28]. This, as Foster [28] points out, limits evaluation in a “normal performance environment” and misrepresents the yearly training process coaches engage in. Single subject research designs would be an alternative, potentially useful approach where athletes can be studied in their training and performance environments over a duration which would make the results more applicable to other coaches/practitioners. Single subject designs have already been recommended to study conditioning in sport [29] with recommendations on how to approach single subject designs research for elite sport recently published [30].

Practically, the aforementioned suggestions highlight the importance of sport science researchers looking to coaches and practitioners to inform their research and coaches integrating such researchers in their community to help identity and address questions pertinent to them. From a researchers standpoint, their efforts in research are (most often) evaluated as being meritorious if they are published within appropriate, high-quality, peer reviewed journals. However, applied research projects are often deemed not meritorious relative to more controlled “exercise science” type research and often criticized for poor sample sizes and non-traditional statistics [31]. This feature of academia may limit motivation to engage in questions that are particularly pertinent to coaches or practitioners specifically. We would suggest that if more peer reviewed applied sport science journals were funded by international sport organizations this may increase engagement in applied research.. There is currently no triathlon specific journal that would promote the dissemination of coach education, psychology, sport management, sport science, and health aspects of triathlon research. Interestingly, a recent book entitled “Triathlon Medicine” [32] provides information that is purported to be the “ultimate clinical guide to all the medical issues related to triathlon”. However, by virtue of this book being published indicates that current state of health and wellness is not good, or at least could be improved, in triathlon. Thus, futureproofing via prospective engagement of relevant research driven by coaches and researchers is still very important and could be better disseminated via more triathlon specific or applied sport science journals.

Furthermore, it is important to highlight how the organizational features of an international organization such as the ITU and national federations affect research engagement. We have not found any research that would guide how change management might occur in this regard. Certainly, most international organizations have some sport science type conference annually or biennially. However, these conferences are for information dissemination primarily, with little time devoted to engagement or organic collaborative research connections between coaches and researchers. We recommend that portions of these conferences be used to develop collaborative research questions and that the ITU and national federations contribute adequate research resources via a research fund that could answer these questions. These authors have also attempted to engage in organic research collaborations with triathlon and endurance coaches via the “Canadian Endurance Coach Network”. This network is driven by the full-time coaches and leaders in triathlon, endurance running and swimming where monthly conference calls focus on a single topical issue in endurance sport as a means to informal learning. The sport scientists on these calls provide the evidence and theory in combination with the experiential comments from coaches to create resolutions and strategies moving forward. As part of the network we have undertaken our first collaborative research project which is a training history, injury and wellness questionnaire designed to evaluate the “state of developmental athletes” in triathlon and other endurance sports. This is a good example of how organic collaborative research networks might be a start in driving more evidence that has immediate utility for coaches and practitioners.

Themes 3 and 4 indicate that a more inclusive approach to athlete management is imperative to improved health, wellness and performance in triathlon. Looking to evidence that would inform themes 3 and 4, Coutts [17] identified “athlete values”, as part of a good evidence-based practice approach to coaching. Specifically, the author recommends incorporating “athlete preferences” into decision making for training and performance, something the panelists also strongly advocated for. Panelists enforced the idea that to move forward as a sport we also need to hear from those who have already left triathlon not on their own accord (i.e. due to injury, illness, or personal reasons). We believe panelists were highlighting two important points; first, that understanding past risk factors will improve coach decision making and second, that including athletes in the decision-making process will reduce risk of injury and dropout. We think this is most important for those coaches who work in youth triathlon where a healthy inclusive coach-athlete relationship is directly related to youth athletes’ continued involvement in the sport [33, 34].

If coaches and practitioners were to improve evidence that is applicable and relevant to triathlon then the information garnered from the panelists and follow up interviews in this project indicate that monitoring tools would be a good place to start. Panelists highlighted the importance of monitoring tools to provide objective criteria for ensuring that athletes do not become over fatigued leading to overtraining [35]. However, studies in the area have shown that coaches’ and sport scientists’ likely place too much reliance on objective data, which affects both the coach-athlete relationship and limits the practicality of some monitoring tools [35, 36]. Thus, engaging in research which examines monitoring tools that are specific to triathlon and also are valid (purports to measure what it was designed to measure) and practical (time, cost, ease of use, not damaging to the athlete) would be beneficial to the future of triathlon sport science. However, as highlighted by the panelists, the emergence of numerous monitoring tools can increasingly complicate the sport, as well as the coach-athlete relationship, particularly if the data ultimately distract from a holistic understanding of the athlete. A recent example of how to utilize objective and subjective measures to determine a more holistic assessment of athlete status has shown promising results [37]. Specifically, these authors tackled “athlete recovery” via a multivariate assessment and found a robust estimation of athlete recovery using a holistic approach [37]. Thus, panelists clearly understood that coaches and practitioners are aware that monitoring data can be useful as long as it does not replace subjective assessments, which have been shown to be useful in understanding athlete status [2, 38]. Further, as discussed above, new research reflects that this combined approach to athlete monitoring is achievable moving forward.

The importance of treating athletes as people not machines was also highlighted in theme four, especially given the expansion of measurement data that can be collected on an athlete, despite the questionable use for most of it [39]. Panelists did not discount the utility of data to improve performance, but rather that understanding the individual athlete as a person, in combination with data gathering, is a worthwhile objective in futureproofing the sport. An underlying cautionary note that is evident from panelists’ opinions of treating athletes like machines, has been echoed by social theorists who have studied the benefits and downsides of it [40]. In this context, endurance athlete coaches often view the athlete’s body as an “engine” that can be tweaked and worked on, not unlike that of a race car [41]. However, as suggested by the panelists, if a positive impact is to be made to athletes’ training and competition practices, coaches must take the time to understand the athletes’ broader lives and how it can impact their health and performance. This reinforces the need for coaches to have an athlete-centered approach to their practice. It has been suggested that an athlete-centered approach requires the definition of clear and mutual performance goals while considering an athletes’ health and lifestyle during the goal setting process [42].

The last theme (theme 5) “challenging accepted cultural and sporting norms” addresses the culture of triathlon where panellists questioned whether some of the spirit of the “initial challenge of triathlon” has been lost [43]. Panellists also questioned whether the present dogma of “doing things because that is the way it’s been done” would futureproof the sport. A good example provided by one panellist was the assessment of body weight where this panellist highlighted that lighter bodies were more efficient despite the known health consequences which can emerge from underweight athletes [44]. However, despite this knowledge regarding body weight health consequences, the panellist highlighted that the “system” has not adequately addressed or advocated for widespread change. In addition, triathlon does not seem to be unique in having entrenched dogma’s which dictate perceptions and actions of coaches and practitioners which lead to health and wellness concerns in triathlon. Research in other sports such as rowing [45] point to a normalization of ill health as part of the sport development process. However, study of system wide abuses or dogmas in other sports has provided few pragmatic solutions which could be used to futureproof triathlon. Thus, this document and the gathering of individuals at the Science and Triathlon 2017 conference are seen as positive steps forward in implementing system wide change and willingness for culture change in triathlon.

Some of this interest in culture change we believe comes from the evidence associated with large numbers of triathletes persevering through pain, injury, burnout, and illness [2, 7, 9, 10] where the accumulated evidence is hard to ignore, stimulating conversation on how to change. Of the limited evidence to assist in culture change we borrow from another serious health issue in sport, concussions. In the context of impact sports where concussions are a serious consequence of sport participation the identification of the issue was the first step to culture change [46]. Thus, we feel that this document is a first step in culture change for triathlon as it relates to triathlete health, welfare and enjoyment.

## Conclusions

This paper serves to address how triathlon as a sport can improve health and also performance in the present and future generations of triathletes. To summarize, we developed 5 themes: 1) critical appraisal and application of knowledge, 2) integrated approaches to developing, disseminating, and using research and expertise, 3) appropriate development and use of measures for monitoring training and recovery, 4) knowing your athletes and adopting holistic approaches to athlete/person-development, 5) challenging accepted cultural and sporting norms.

The first theme highlights that critical appraisal reduces bias and improves reflective practice. In the second theme a renewed focus on developing greater triathlon specific evidence and connecting current evidence to inform practice was highlighted. The third theme acknowledges that triathlon requires some new approaches to healthy development to reduce the incidence of overtraining and injury. The fourth theme recognizes the importance of the triathlete and that to improve triathlete health and performance an investment in an athlete centered approach is necessary. The last theme, challenging accepted cultures indicates a willingness to move the sport of triathlon forward by examining other ways of “doing things”. However, the development of these themes is only the first step in futureproofing the sport because to effect change, resources and coordination are required from the International Triathlon Union and the collective federations. We propose that the ITU Grand Finals (August 2020) be a key part of any future implementation of these recommendations. All the world federations will be represented as well as the head ITU policy makers at the Grand Final, thus a collective dissemination of these recommendations could occur. Furthermore, to evaluate whether change has occurred future Science of Triathlon conferences (South Africa 2021) should hold additional roundtables using these recommendations as the thematic discussion areas. The authors of this document are working with the next Science of Triathlon conference organizers to ensure that “futureproofing sessions” will occur as well. However, any event that brings together athletes, coaches, practitioners, academics and policy makers to debate and share their own successes and challenges will likely improve the culture of triathlon thereby futureproofing triathlon. It is additionally evident that there is good current awareness of the issues and also a willingness to act on the futureproof themes within the global triathlon community. Thus, we hope this paper aids in improving health and performance in triathletes worldwide.

## Data Availability

In keeping with regulations of the Health Research Board at the University of Alberta restrictions apply to the availability of interview data to ensure confidentiality and privacy of all participants. The anonymized datasets used and analyzed for the current study are available from the corresponding author on request due to privacy restrictions.

## Abbreviations

ITU: International Triathlon Union
VO2max: Maximal Oxygen Consumption
